# Treatment of Restless Legs Syndrome Improves Agitation and Sleep in Persons with Dementia: A Randomized Trial

**DOI:** 10.1016/j.jamda.2025.105485

**Published:** 2025-03-31

**Authors:** Kathy C. Richards, Liam M. Fry, Alicia J. Lozano, Wenyan Ji, Janet D. Morrison, Katherine C. Britt, Donald L. Bliwise, Nalaka S. Gooneratne, Alexandra L. Hanlon

**Affiliations:** a School of Nursing, University of Texas at Austin, Austin, TX, USA; b Department of Internal Medicine and Austin Geriatrics Specialists, Dell Medical School, University of Texas at Austin, Austin, TX, USA; c Department of Statistics, Virginia Tech, Roanoke, VA, USA; d College of Nursing, University of Iowa, Iowa City, IA, USA; e Department of Neurology, Nell Hodgson Woodruff School of Nursing, Emory University, Atlanta, GA, USA; f Perelman School of Medicine at the University of Pennsylvania, Philadelphia, PA, USA

**Keywords:** Nighttime agitation, restless legs syndrome, dementia, Alzheimer’s disease, gabapentin enacarbil, sundowning

## Abstract

**Objectives:**

Restless legs syndrome (RLS), a common, treatable, sensorimotor disorder of nighttime uncomfortable leg sensations that interfere with sleep, may prompt nighttime agitation in persons with dementia.

**Design:**

This randomized trial was double-blind and placebo-controlled. Participants received a Food and Drug Administrationeapproved drug for RLS, gabapentin enacarbil (GEn) (Horizant) or placebo.

**Setting and Participants:**

Older adults (N = 147) with dementia due to Alzheimer’s disease, nighttime agitation, and RLS, residing in long-term care or at home, participated.

**Methods:**

The primary outcome was change from baseline to 8 weeks in nighttime agitation between 5 pm and 7 am on the Cohen-Mansfield Agitation Inventory Index, Direct Observation. Multivariable linear mixed effects regression models based on multiply imputed data were estimated on nighttime agitation and sleep, with treatment group, week, the 2-way interaction of group and week as predictors, and mean arterial pressure as a covariate based on baseline group imbalances.

**Results:**

Mean age ± SD was 83.4 ± 9.1 years. Most were female (72.0%), white (92.3%), non-Hispanic (84.6%), and lived in nursing homes (76.9%). Nighttime agitation, by group over time, was significant at 8 weeks (estimate, −1.67; *P* = .003) and 2 weeks. Total sleep time (actigraphy) by group over time was significant at 8 weeks (estimate, 48.45; *P* = .026). Observed nighttime wake by group over time was significant at 2 (estimate, −12.54; *P* = .006) and 8 weeks (estimate, −11.12; *P* = .015). The number having ≥1 adverse events was 60 in the GEn group (81.1%) and 50 in the placebo group (68.5%); with 12 serious adverse events in placebo and 10 in the GEn group. The GEn group had a trend toward more falls (*P* = .066).

**Conclusions and Implications:**

Our findings suggest a novel approach for nighttime agitation in persons with dementia: assessing for RLS and initiating interventions. Larger and longer trials are needed.

Neuropsychiatric symptoms in persons with dementia due to Alzheimer’s disease (AD-D) are distressful for caregivers and challenging to manage. One prevalent symptom is nighttime agitation, defined as the presence or worsening of behavioral symptoms at night (eg, emotional distress, loud vocalizations, wandering, physically aggressive behaviors).^1,2^ Nighttime agitation may overlap with sundowning symptoms in the late afternoon and early evening.

Aligned with precision health initiatives, we began studies to explore whether nighttime agitation could represent manifestation of a sensorimotor condition, restless legs syndrome (RLS). RLS is characterized by an urgent need to move the legs and strange and uncomfortable leg sensations in the first part of the night.^3^ Symptoms closely mirror those of nighttime agitation (eg, restlessness) and a circadian tendency to occur in the evening. In AD-D, the challenge lies in their diminished ability to verbally express and relieve their RLS discomfort. As a result, their distress may manifest as nighttime agitation. Iron therapy, deprescribing medications that worsen symptoms, and Food and Drug Administration–approved medications are effective for RLS and could potentially reduce nighttime agitation.^4^

RLS often remains undiagnosed in AD-D because their cognitive and language deficits prevent them from reporting the specific symptoms for the gold standard RLS diagnosis.^5^ In earlier work, our team addressed unreliable RLS diagnosis in AD-D by using a combination of indicators including polysomnography, caregiver interviews, iron levels, observation for RLS-associated behaviors, and expert consensus. With these comprehensive methods, we found that about one-fourth of older adults with dementia and nighttime agitation had RLS and that RLS was associated with nighttime agitation (*r* = 0.31, *P* = .01).^6^ Next, we created and validated a new diagnostic instrument feasible for RLS diagnosis in AD-D, the Behavioral Indicators Test Restless Legs (BIT-RL).^7^

In this trial, we aimed to understand if RLS treatment with a Food and Drug Administration–approved medication for RLS, gabapentin enacarbil (GEn) (Horizant), reduced nighttime agitation. The prespecified hypotheses were as follows: (1) compared with the placebo group, the GEn treatment group will have fewer nighttime (5 pm to 7 am) agitation behaviors (primary outcome), better sleep, and fewer RLS behaviors (secondary outcomes); and (2) frequency of RLS behaviors will mediate the effect of GEn on nighttime agitation (secondary outcome).

## Methods

### Design

The protocol for this trial has been reported.^8^ This study was an 8-week, double-blind, placebo-controlled randomized trial of GEn vs placebo. Outcomes were measured at baseline, 2 weeks, and 8 weeks. Adverse events (AEs) were measured during the trial and 8 weeks posttrial.

### Participants

Participants were recruited from 31 long-term care homes and independent living. Eligibility criteria and exclusions are listed in Table 1.

### RLS Diagnosis

The BIT-RL consists of clinical and behavioral indicators.^7^ The study nurse obtained the clinical indicators (nonspecific leg discomfort, difficulty falling asleep, daytime fatigue, history of iron deficiency, RLS family history, and diabetes) from caregivers, the medical record, and participants (if able). Research assistants (RAs) observed participants for 20 minutes between 5 pm and scheduled bedtime, generating 10 ratings every 2 minutes for RLS behavioral indicators (using hand to hold/rub leg or foot, rubbing legs or feet together, kicking, pushing against a surface, moving as if pushing on a gas pedal, stretching/straightening, crossing and uncrossing legs or feet, and fidgeting). The RAs were trained by investigator K.C.R., and they reached and retained an interrater reliability of 90%. Diagnostic sensitivity of the BIT-RL is 78% and specificity is 78%, with 77% correctly classified.^7^ The total score ranges from 0 to 16 (clinical indicators: 0–6, behavioral indicators: 0–10). The medical team, consisting of investigators L.M.F., N.S.G., and Richard P. Allen, made the RLS diagnosis using the criterion of total BIT-RL score >6 and behavioral indicators score >3.

### Ethics

The study was approved by the institutional review board (trial registration: Clinical Trials.gov Identifier: NCT03082755, date of registration March 6, 2017). Legally authorized representatives and participants (if able) gave written informed consent. The investigators designed the trial, analyzed the data, and wrote the manuscript. Azurity Pharmaceuticals Inc markets Horizant, and Arbor Pharmaceuticals LLC provided the GEn and placebo. They did not influence the design, conduct, or publication of the trial.

### Interventions

Participants were randomized to either GEn or matching placebo. GEn has a good safety profile and significant differences when compared with placebo on RLS symptoms and sleep.^3,4,9,10^ The placebo group received identical inactive capsules. The medical team prescribed the dosage, monitored response, and made treatment decisions.

The starting dose was 1 capsule (300 mg GEn or placebo) with food at 5 pm. The medical team reviewed response after 2 weeks to determine if each participant should continue or increase to 2 capsules. Dosage was based on the creatinine clearance (CC) using GEn prescribing guidelines: CC ≥60 mL/min: 600 mg daily; CC 30–59 mL/min: 300 mg daily and increase to 600 mg as needed; CC: 15–29 mL/min: 300 mg daily,^9^ and participant response. The facility staff or home caregivers administered the capsules. The study nurses reviewed the medication records and dose packages.

### Baseline Measures

Study nurses collected baseline characteristics, age, sex, race, ethnicity, education, and current medications from the electronic health record. They used the Cumulative Illness Rating Scale – Geriatrics (0–4) to assess physical impairment.^11^ They measured height and weight, measured resting mean arterial blood pressure (MAP), and drew fasting samples for ferritin and CC. Caregivers reported ambulation status, snoring, nonpharmacologic interventions, and dizziness.

### Outcomes

The prespecified primary outcome was change from baseline to 8 weeks in nighttime agitation between 5 pm and 7am, measured by the 14-item Cohen Mansfield Agitation Inventory (CMAI) Index, modified for continuous direct observation.^8,12–14^ Trained RAs continuously observed residents for agitation behaviors using an infrared camera and remote monitor. The modified CMAI Index requires that the RAs first note whether the participant is behaviorally awake or asleep. The RAs recorded behaviors every 5 minutes during wake. The interrater reliability of the RAs for detecting sleep, wake, and agitation with an expert and each other in our earlier work ranged from 0.80 to 0.95.^6^ In this trial, the RAs achieved and maintained agreement of *r* > 0.90 with the investigator and each other.^8^ The primary outcome, the CMAI Index (range, 0–14), is the number of behaviors divided by the number of hours observed. The observations occurred from 5 pm to 10 pm on one night and 10 pm to 7 am on a second night at baseline and intervention weeks 2 and 8. In additional analyses, we also examined treatment effects at 2 weeks and by different observation periods. Other prespecified key secondary outcomes were sleep duration from 10 pm to 7 am as measured by 3–7 days and nights (as tolerated) of wrist actigraphy (Ambulatory Monitoring Inc or Mini-Mitter Inc), number of awakenings as measured by the CMAI Index trained observers, and frequency of RLS behaviors as measured by the raw score of the behavioral indicators section of the BIT-RL.^7^ Other prespecified outcomes were the CMAI (Caregiver Version),^14^ and the Modified Alzheimer’s Disease Cooperative Study-Clinical Global Impression of Change.^15^ Safety was assessed by a study nurse using an AE checklist, physical examination, caregiver reports, and the medical record.

### Sample Size and Power

Power was determined a priori through 100 simulations for the primary outcome, nighttime agitation, using the primary analytical approach of a linear mixed effects model to evaluate changes over time between the 2 groups. A sample size of 136 (68 per group) achieved over 90% power to detect a minimum detectable difference of 0.25 in changes in the CMAI Index over time, reflected in the interaction term (group × time), assuming a 2-sided alpha of 0.05. This calculation was based on an assumed mean baseline CMAI Index ± SD of 1.5 ± 0.90 in both groups, a within-subject variance of 1.6, an autocorrelation coefficient of 0.81, and expected mean CMAI Index values at 8 weeks of 1.35 in the placebo group (representing a 10% reduction) and 1.0 in the GEn group. To account for dropouts during the COVID-19 pandemic, the sample size goal was increased to 147. No interim analyses were planned or performed.

### Randomization and Masking

A statistician, uninvolved in data management or analysis, created the randomization system, by site, by A or B group. In this double-blind study, only the research pharmacist was unblinded to treatment.

### Statistical Methods

All analyses were specified a priori and performed in the intention-to-treat population, defined as analyzing the data according to the treatment group participants were assigned. Baseline characteristics (Table 2) were summarized, and treatment groups were compared. Multiple imputation by chained equations was used to address missing data (<20% was missing) for analyses of the primary and secondary outcomes of nighttime agitation, wake and sleep (observed), and RLS behaviors after excluding 4 participants missing MAP data.^16,17^ The primary and secondary analyses relied on multivariable linear mixed effects regression models based on multiply imputed data. Models were estimated for nighttime agitation, wake and sleep (observed by RA), and RLS behaviors, with treatment group, week, the 2-way interaction of treatment group, and week (group × week) included as predictors and MAP included as a covariate based on baseline group imbalances. Parameter estimates, 95% CIs, *P* values, and mean predicted plots at each time point by treatment group were reported. Because missing actigraphy data was >40%, analyses were based on the observed data.^18^ Multiple imputation was conducted using the mice package in R. All other analyses were performed using SAS V9.4 (SAS Institute Inc). Statistical significance was taken at the 0.05 level and did not account for multiplicity due to the pilot nature of the study.

## Results

### Recruitment

Participants were recruited from July 2017 until June 2022. Final assessments were completed on August 1, 2022, and the study code was broken shortly thereafter.

### Participant Flow

**Fig. 1.**
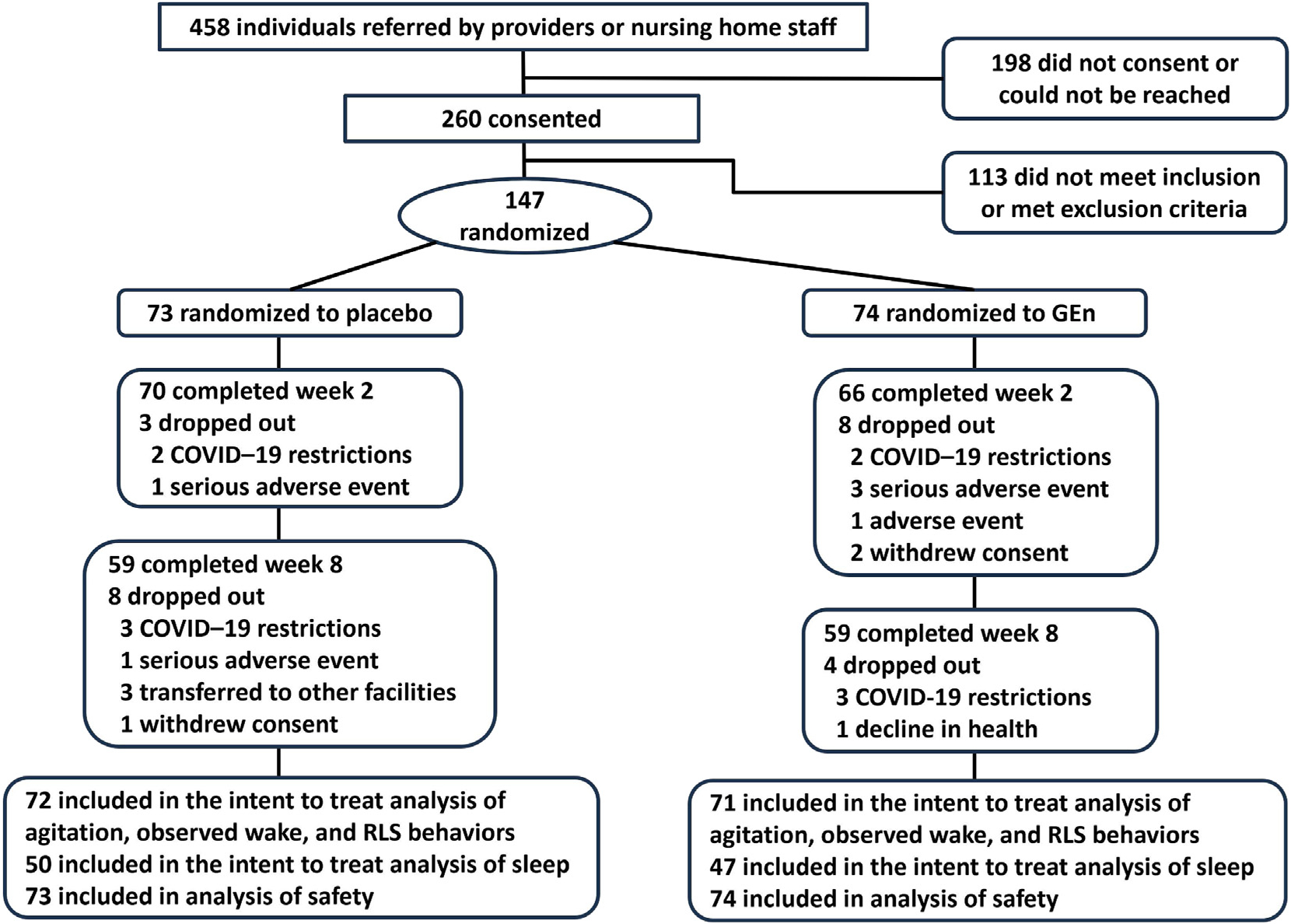
Participant flow.

A total of 458 individuals were referred, 260 consented, and 147 were randomized, with 74 (50.3%) in the GEn group and 73 (49.7%) in the placebo group (Figure 1). Number of dropouts was 11 in the placebo group and 12 in the GEn group. Of the 210 participants who met the criteria for RLS, 5 had a previous RLS diagnosis (2.4%).

### Baseline Data

Baseline characteristics are summarized in Table 2. Participants in both groups were receiving sedating medications for behavioral and sleep disturbances (eg, anticonvulsants, antidepressants, antihistamines, antipsychotics, sedative-hypnotics). The only significant baseline difference between groups was in blood pressure (Table 2). Specifically, those in the placebo group had a higher MAP compared with the GEn group (95.7 vs 92.0, *P* = .023). Thus, we controlled for MAP in analyses. Four individuals missing MAPs were excluded. Therefore, 143 was the sample size for analyses of nighttime agitation, observed wake, and RLS behaviors. The sample size for actigraphy sleep data was 97 due to participants who refused or removed the actigraph. AEs were monitored in all 147 randomized individuals.

### Primary Outcome

**Fig. 2.**
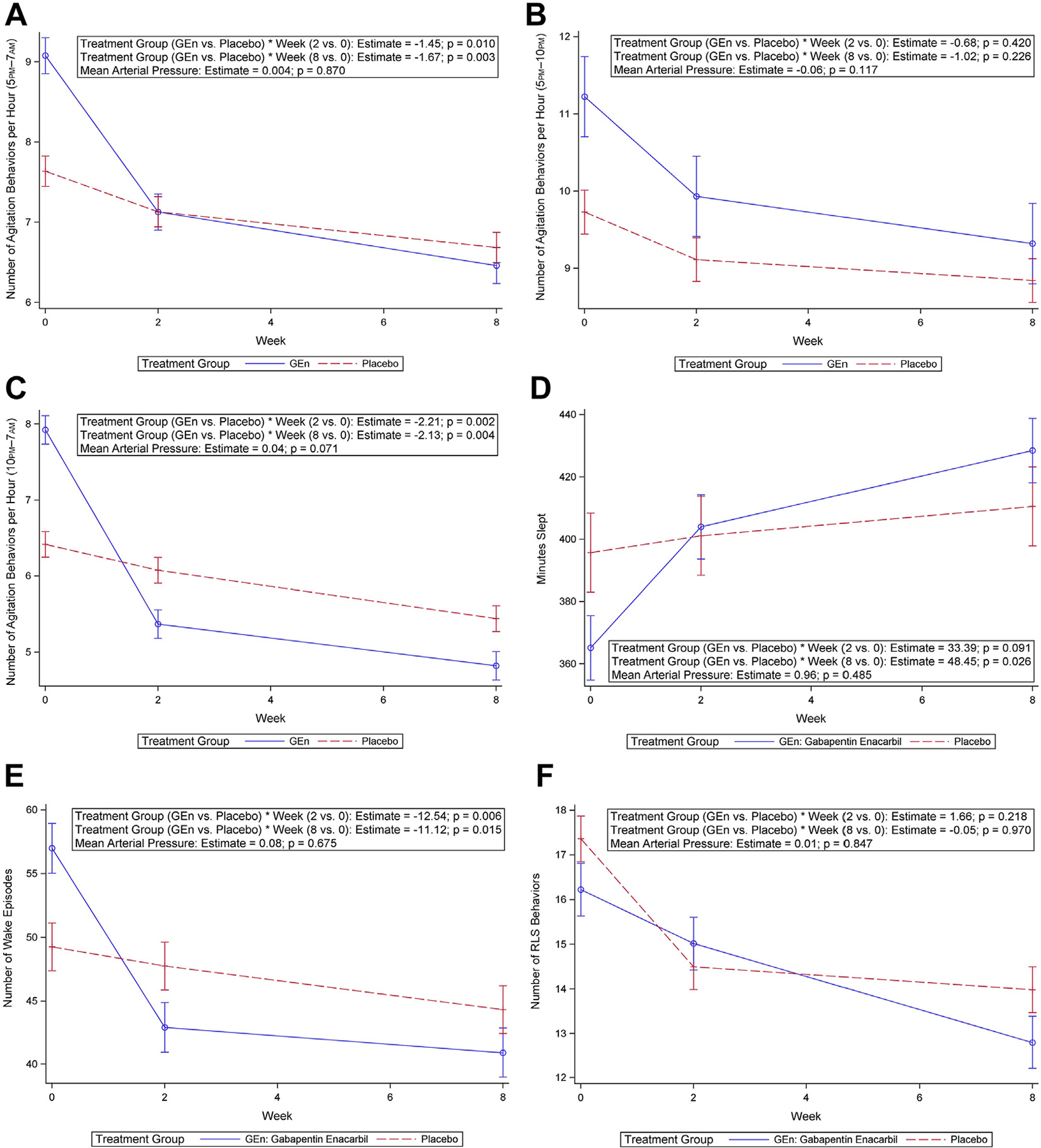
Predicted plots by treatment group of outcomes. (A) Nighttime agitation behaviors (5 pm–7 am). (B) Nighttime agitation behaviors (5 pm–10 pm). (C) Nighttime agitation behaviors (10 pm–7 am). (D) Total nighttime sleep. (E) Observed nighttime wake. (F) RLS behaviors.

The primary outcome, nighttime agitation (5 pm to 7 am) by group over time, controlling for MAP, was statistically significant at 8 weeks (estimate, −1.67; *P* = .003) (Figure 2A; Supplementary Table 1). In secondary analyses, we examined treatment effects at 2 weeks and by different times of night. Nighttime agitation by group over time, controlling for MAP, was statistically significant at 2 weeks (Figure 2A). There were no significant changes in agitation from 5 pm to 10 pm by group over time, controlling for MAP, at either 2 or 8 weeks (Figure 2B; Supplementary Table 1). Statistically significant changes from 10 pm to 7 am in agitation by group over time, controlling for MAP, were observed at 2 weeks (estimate, −2.21; *P* = .002) and 8 weeks (estimate, −2.13; *P* = .004) (Figure 2C, Supplementary Table 1). These improvements were clinically meaningful,^19^ with 32.2% and 39.1% reductions in agitation from baseline at 2 and 8 weeks (Table 3).

### Secondary Outcomes

The predicted plot by group on total nighttime sleep by group over time was significant at 8 weeks (estimate, 48.45; *P* = .026). (Figure 2D; Supplementary Table 2). Improvements in the GEn group were clinically meaningful,^20^ with 61.14 more minutes slept at 8 weeks, compared with baseline (Table 3). The placebo group slept 6.78 fewer minutes at week 2 and 18.25 more minutes at week 8 compared with baseline. Changes in observed nighttime wake by group over time, controlling for MAP, were significant at 2 weeks (estimate, −12.54; *P* = .006) and 8 weeks (estimate, −11.12; *P* =.015) (Figure 2E; Supplementary Table 2). Improvements in number of observed wake episodes in the GEn group were clinically meaningful,^20^ with 24.7% and 28.2% fewer wake episodes at 2 and 8 weeks (Table 3).

No significant changes in RLS behaviors, controlling for MAP, were observed at 2 weeks (estimate, 1.66; *P* = .218) or 8 weeks (estimate, −0.05; *P* = .970) (Supplementary Table 3, Figure 2F). The mediating effects of frequency of RLS behaviors on the relationship between GEn and nighttime agitation were examined using a modified Baron and Kenny approach.^21^ Although significant associations between treatment group and nighttime agitation over time (group × week, *P* = .003) were observed, there was no significant relationship between treatment group and RLS behaviors over time (group × week, *P* = .353) (data not shown).^21,22^

There was no significant difference between groups on the CMAI Caregiver Version or the modified Modified Alzheimer’s Disease Cooperative Study-Clinical Global Impression of Change (Supplementary Table 3, Table 3). In the sensitivity analysis excluding those with medication changes during the trial (n = 70), some of the significant findings became nonsignificant, but the directions of the estimates did not change. There were no differences by race, sex, or ethnicity in the study outcomes (data not shown).

### Safety

Serious adverse events (SAEs), including deaths, were evenly distributed between groups (Table 4). There were 22 SAEs, with 12 in placebo (4 possibly related), and 10 in GEn (5 possibly related). Two deaths in placebo and 1 death in GEn were possibly related. The number having ≥1 AE was 110 (74.8%), with 60 (81.1%) in the GEn and 50 (68.5%) in placebo (Table 4). Events experienced by ≥10% of participants were falls, somnolence/sedation (GEn: 16.2%; placebo: 2.7%), and irritability (GEn: 13.5%; placebo: 12.3%). Thirty-six participants (51%) had 60 falls possibly related to GEn (data not shown). Supplementary Table 4 shows fall incidence.

The study data safety monitoring board (DSMB) met on July 12, 2022. The DSMB reviewed descriptive data during their closed sessions (eg, number of falls) stratified by blinded A/B groups. Due to their concerns about falls and sedation, the DSMB requested unblinded descriptive AE data. The data showed that the GEn group had more falls and somnolence/sedation than the placebo group. Given the AE data and the project having exceeded the original sample size goal, the DSMB voted to stop the study drug or placebo in the 6 remaining participants. The DSMB did not have other concerns. They recommended that other aspects of the project continue.

After data collection was completed and the study blind broken, the inferential analysis showed that the mean number of falls ± SD per participant was 1.0 ± 1.6, with the GEn group having more falls than the placebo group (mean, 1.3 vs 0.7; *P* = .030) (Supplementary Table 5). After adjusting for baseline MAP, there was no statistical difference in number of falls between groups (odds ratio, 1.89; 95% CI, 0.096–3.71; *P* = .066).

## Discussion

In older adults with AD-D, RLS, and nighttime agitation, RLS treatment with GEn, compared with placebo, resulted in statistically significant improvements over time in the primary study end point (nighttime agitation) and key end points (total nighttime sleep and number of wake episodes). In analysis by observation period, treatment with GEn compared with placebo resulted in statistically significant and clinically meaningful improvements in agitation from 10 pm to 7 am. However, it did not significantly reduce agitation from 5 pm to 10 pm or the frequency of RLS behaviors. These findings may be related to the timing of GEn administration at 5 pm and the potential for delayed absorption in an older population.^23^ Although this study was not designed to assess dosage, a 300-mg dose of GEn appeared to be effective.

The incidence of AEs in the GEn group was 81% vs 69% in the placebo group. In a recent trial on brexpiprazole for agitation, 40.7% in the treatment group and 31% in the placebo group reported treatment-emergent AEs.^24^ The severity and seriousness of the AEs did not differ between groups in the current trial; however, the incidence of severe AEs and SAEs was relatively low. Falls were the most common AE, and they were more likely in the GEn group. Almost 60% of the GEn group fell, compared with about 41% of the placebo group. Differences in number of falls were not statistically significant after controlling for MAP. Future larger studies might further examine the impact of comorbidities (eg, blood pressure) on the overall safety of GEn in this population.

Although nonpharmacologic interventions are preferred for behavioral symptoms, not all patients respond,^1^ and evidence on the efficacy and feasibility of nonpharmacologic interventions for RLS is lacking.^11,12^ One interesting aspect of this study is the improvements in agitation and sleep in the placebo group, perhaps indicating functional expectation pathways and the impacts of supportive, empathetic care by research staff on hope and response.^25^

RLS affects about 7% of adults, with most studies showing a higher prevalence in females and older adults.^26,27^ In our earlier work, 24% of older adults with dementia and nighttime agitation had RLS.^6^ Although our data do not reflect RLS prevalence, about 95% of those screened met criteria for RLS. Several factors likely contributed. First, everyone in this study had nighttime agitation, and our results have confirmed that RLS is a cause for this problem. Second, older adults with dementia frequently have RLS risk factors (eg, age, iron deficiency, consumption of medications that worsen or cause RLS). Also, it is possible that the study medical team overdiagnosed RLS; however, our significant GEn group treatment effects do not support this. Another contributing factor might be the study referral processes. The providers and caregivers were aware that the study focused on RLS, and they may have referred those likely to have RLS or previously diagnosed with RLS.

A limitation is this trial was conducted during and after the COVID-19 pandemic. Staffing shortages and turnover hindered collection of clinician’s impressions of change and caregiver ratings of nighttime agitation.^14,15^ Fall frequency may partly reflect incidents when older adults needed assistance but could not obtain it. Alternately, RLS, sleep disturbances, or nighttime agitation may increase fall risk, and additional research is warranted to examine this idea. Few falls had serious consequences, a positive finding regarding successful injury prevention strategies.

## Conclusions and Implications

This trial highlights RLS as a significant, yet often overlooked, cause for nighttime agitation and sleep disturbance in AD-D. Addressing RLS through deprescribing medications that worsen symptoms, treating iron deficiency, and carefully weighing pharmacologic options offers a novel approach. Further research is needed on effective nonpharmacologic interventions and larger, long-term studies.

## Supplementary Material

1

Supplementary data related to this article can be found online at https://doi.org/10.1016/j.jamda.2025.105485.

## Figures and Tables

**Table 1:** Eligibility Criteria and Exclusions

Eligibility Criteria	Exclusions
>55 years of age Clinical Dementia Rating 0.5–3 Physician diagnosis of Alzheimer’s dementia Nighttime agitation, defined as Cohen-Mansfield Agitation Inventory, Direct Observation total score >35 Medication for agitation was appropriate Medically stable Can swallow uncrushed medications Ambulatory with or without assistance If being treated for RLS, persistence of RLS symptoms/signs RLS diagnosis using the Behavioral Indicators Test-Restless Legs	Received ≥50 morphine milligram equivalents daily during the past 14 days Receiving gabapentin or GEn Tremor Severe psychosis Alcohol consumption GEn contraindicated Failed past treatment with gabapentin or GEn Creatinine clearance <15 mL/min or hemodialysis Participation in any study that may affect outcomes Suicide risk Any condition making enrollment inappropriate Community-dwelling without a caregiver

**Table 2:** Baseline Characteristics of 143 Older Adults With Dementia Due to AD-D and RLS Randomly Allocated to GEn or Placebo

Characteristic	GEn (n = 71)	Placebo (n = 72)	*P* Value*
Age, y			
Mean ± SD	83.69 ± 8.30	83.19 ± 9.92	.747
Sex			
Female	49 (69.0)	54 (75.0)	.425
Race^†^			
Black	2 (2.8)	6 (8.3)	.452
White	67 (94.4)	65 (90.3)	
Other	2 (2.8)	1 (1.4)	
Ethnicity			
Hispanic or Latino	13 (18.3)	9 (12.5)	.336
Education			
High school or less	36 (50.7)	43 (59.7)	.278
Level of care			
Skilled nursing care	56 (78.9)	54 (75.0)	.868
Assisted living	11 (15.5)	13 (18.1)	
Independent living	4 (5.6)	5 (6.9)	
Ambulation			
Ambulatory with assistance	53 (74.6)	52 (72.2)	.743
Ambulatory without assistance	18 (25.4)	20 (27.8)	
CIRS-G, severity index^‡^			
Mean ± SD	1.84 ± 0.28	1.86 ± 0.29	.791
Body mass index, kg/m^2^			
Mean ± SD	25.61 ± 5.88	25.57 ± 5.14	.834
MAP			
Mean ± SD	91.99 ± 10.20	95.70 ± 9.05	.023
Ferritin, ng/mL^§^			
Mean ± SD	129.38 ± 125.74	126.50 ± 118.19	.969
CC, mL/min^||^			
Mean ± SD	62.48 ± 24.48	59.76 ± 28.46	.291
History of dizziness			
Yes	4 (5.6)	1 (1.4)	.209
Snore			
Yes	19 (26.8)	16 (22.2)	.528
Nonpharmacologic interventions used for nighttime agitation			
None	35 (49.3)	30 (41.7)	.632
Communication	20 (28.2)	19 (26.4)	
Activity	9 (12.7)	12 (16.7)	
Other	7 (9.9)	11 (15.3)	
Medications			
AD-D	30 (42.3)	31 (43.1)	.923
Analgesic	63 (88.7)	66 (91.7)	.555
Anticonvulsant	12 (16.9)	16 (22.2)	.423
Antidepressant	47 (66.2)	49 (68.1)	.813
Antihistamine	29 (40.8)	22 (30.6)	.199
Antipsychotic	9 (12.7)	18 (25.0)	.060
Anxiolytic	8 (11.3)	7 (9.7)	.763
Beta2 agonist	15 (21.1)	11 (15.3)	.365
Beta blocker	24 (33.8)	22 (30.6)	.678
Opioid	10 (14.1)	10 (13.9)	.973
Sedative/hypnotic	30 (42.3)	30 (41.7)	.943
Other	69 (97.2)	71 (98.6)	.620

CIRS-G, Cumulative Illness Rating Scale-Geriatrics.

Values are n (%) or as otherwise indicated.

* *P* values are based on 2-sample *t* tests or Wilcoxon rank sum tests or Fisher exact tests or χ^2^ tests, as appropriate.

† For race, other is a combination of Asian and Native Hawaiian/Pacific Islander.

‡ CIRS-G range 0–4.

§ Serum ferritin range 20–500 ng/mL.

|| CC range 90–140 mL/min.

**Table 3:** Outcomes by Group at Baseline, 2 Weeks, and 8 Weeks With Imputed Data or Complete Case Analysis

Variable	Baseline		2 Weeks		8 Weeks	
	GEn (n = 71)	Placebo (n = 72)	GEn (n = 71)	Placebo (n = 72)	GEn (n = 71)	Placebo (n = 72)
Nighttime and evening agitation						
CMAI 5 pm to 10 pm	52.23 ± 31.80	45.03 ± 19.25	47.63 ± 29.39	41.81 ± 18.97	41.89 ± 26.30	40.86 ± 19.26
CMAI 10 pm to 7 am	63.69 ± 41.56	51.36 ± 29.38	43.48 ± 30.81	48.89 ± 29.32	39.26 ± 24.88	43.16 ± 24.31
CMAI total	115.92 ± 53.98	96.39 ± 35.71	91.10 ± 41.84	90.70 ± 36.85	81.16 ± 37.98	84.03 ± 35.97
CMAI index 5 pm to 10 pm*	11.22 ± 6.92	9.73 ± 4.20	9.93 ± 6.37	9.11 ± 3.98	9.32 ± 5.70	8.84 ± 4.15
CMAI index 10 pm to 7 am	7.92 ± 4.89	6.42 ± 3.59	5.37 ± 3.58	6.08 ± 3.62	4.82 ± 3.02	5.44 ± 3.02
CMAI total index 5 pm to 7 am	9.08 ± 4.04	7.64 ± 2.84	7.13 ± 3.20	7.13 ± 2.95	6.46 ± 3.04	6.68 ± 2.82
CMAI caregiver^†^	30.03 ± 10.80	30.18 ± 11.34	27.30 ± 11.96	30.29 ± 13.18	27.77 ± 10.29	28.28 ± 13.06
Clinical impression change						
mADCS-CGIC improve	–	–	20 (28.2)	27 (37.5)	32 (45.1)	25 (34.7)
mADCS-CGIC, no change	–	–	48 (67.6)	44 (61.1)	31 (43.7)	44 (61.1)
mADCS -CGIC, worsening	–	–	3 (4.2)	1 (1.4)	8 (11.3)	3 (4.2)
Sleep^‡^						
Total nighttime sleep, min	365.75 ± 137.06 (n = 47)	396.14 ± 144.10 (n = 50)	408.21 ± 116.43 (n = 38)	389.36 ± 141.45 (n = 40)	426.89 ± 122.14 (n = 29)	414.39 ± 143.12 (n = 33)
Wake after sleep onset, min	167.84 ± 79.29 (n = 47)	184.81 ± 92.16 (n = 50)	145.19 ± 88.10 (n = 38)	169.46 ± 93.23 (n = 40)	155.95 ± 79.17 (n = 29)	172.24 ± 83.23 (n = 33)
Number of wake episodes observed	56.99 ± 29.89	49.26 ± 26.60	42.93 ± 27.44	47.75 ± 25.99	40.94 ± 25.37	44.34 ± 26.63
Nighttime sleep efficiency, %	50.74 ± 19.02 (n = 47)	54.96 ± 20.00 (n = 50)	56.63 ± 16.15 (n = 38)	54.02 ± 19.63 (n = 40)	59.22 ± 16.95 (n = 29)	57.49 ± 19.86 (n = 33)
Daytime sleep, min	164.50 ± 116.62 (n = 47)	141.53 ± 98.23 (n = 50)	153.32 ± 115.11 (n = 38)	151.03 ± 110.56 (n = 40)	184.09 ± 133.82 (n = 29)	152.62 ± 116.00 (n = 33)
BIT-RL						
RLS behaviors (raw score)^§^	16.23 ± 7.91	17.36 ± 8.44	15.02 ± 8.54	14.50 ± 6.81	12.80 ± 7.96	13.98 ± 7.63
RLS behavioral indicators^||^	8.69 ± 1.86	8.76 ± 1.81	8.30 ± 2.63	8.36 ± 2.17	7.48 ± 2.72	7.84 ± 2.76
RLS clinical indicators**	3.18 ± 0.83	3.15 ± 0.87	1.47 ± 0.68	1.43 ± 0.62	1.28 ± 0.58	1.29 ± 0.77

mADCS-CGIC, Modified Alzheimer’s Disease Cooperative Study-Clinical Global Impression of Change.

Values are mean ± SD or n (%).

* CMAI Index range 0–168 per hour of observation.

† CMAI caregiver range 14–98.

‡ For actigraphy variables total nighttime sleep, wake after sleep onset, nighttime sleep efficiency, and daytime sleep, the descriptive statistics were summarized based on complete case analysis.

§ RLS Behaviors range 0–80.

|| RLS behavioral indicators range 0–10.

** RLS clinical indicators range 0–6.

**Table 4:** Incidence of AEs, SAEs, and Deaths

Incidence of Events* by Participant Number Having ≥1 Event/(Number Randomized = 147)	Total	
	110 (74.8)	
Incidence of Events* by Group Number Having ≥1 Event/(Number Randomized GEn = 74, Placebo = 73)		
Group	GEn	Placebo
Overall^†^	60 (81.1)	50 (68.5)
Somnolence/sedation	12 (16.2)	2 (2.7)
Irritability	10 (13.5)	9 (12.3)
Falls	44 (59.5)	30 (41.1)
Other (ie, urinary tract infection, cataract surgery)	48 (64.9)	46 (63.0)
SAE^‡^	10 (13.5)	12 (16.4)
Injury due to fall		
Hip fracture	1 (1.4)	1 (1.4)
Lumbar 2 fracture	1 (1.4)	0 (0)
Intracranial hemorrhage	0 (0)	1 (1.4)
Subarachnoid hemorrhage	1 (1.4)	0 (0)
Decreased function, mobility resulted in fall	1 (1.4)	0 (0)
Skin infection	0 (0)	1 (1.4)
Cerebrovascular accident	1 (1.4)	0 (0)
Escalating agitation	0 (0)	1 (1.4)
Irregular, rapid heart rate	0 (0)	1 (1.4)
Diabetic ketoacidosis	1 (1.4)	0 (0)
COVID-19	1 (1.4)	0 (0)
Urinary tract infection	1 (1.4)	1 (1.4)
Decline, unknown cause	0 (0)	1 (1.4)
Pancreatic cancer	0 (0)	1 (1.4)
Tachycardia	0 (0)	1 (1.4)
Dehydration	1 (1.4)	1 (1.4)
Bronchitis	0 (0)	1 (1.4)
Right lung mass with pleural effusion	1 (1.4)	0 (0)
Possible pneumonia	0 (0)	1 (1.4)
Deaths^‡^	4 (5.4)	4 (5.5)

* For the overall row, the numerator is the number of participants with at least 1 event.

† Incidence of AEs experienced by at least 10% of participants.

‡ All incidents of SAEs and deaths are reported.

## References

[1] Porsteinsson AP, Antonsdottir IM. An update on the advancements in the treatment of agitation in Alzheimer’s disease. Expert Opin Pharmacother. 2017; 18:611–620. 28300462 10.1080/14656566.2017.1307340

[2] Hjetland GJ, Pallesen S, Thun E, Light interventions and sleep, circadian, behavioral, and psychological disturbances in dementia: a systematic review of methods and outcomes. Sleep Med Rev. 2020;52:101310. 32289734 10.1016/j.smrv.2020.101310

[3] Winkelman JW, Berkowski JA, DelRosso LM, Treatment of restless legs syndrome and periodic limb movement disorder: an American Academy of Sleep Medicine systematic review, meta-analysis, and GRADE assessment-Treatment of restless legs syndrome and periodic limb movement disorder. J Clin Sleep Med. 2025;21:153–199. 39324664 10.5664/jcsm.11392 PMC11701280

[4] Winkelman JW, Berkowski JA, DelRosso LM, Treatment of restless legs syndrome and periodic limb movement disorder: an American Academy of Sleep Medicine clinical practice guideline. J Clin Sleep Med. 2025;21:137–152. 39324694 10.5664/jcsm.11390 PMC11701286

[5] Richards K, Shue VM, Beck CK, Restless legs syndrome risk factors, behaviors, and diagnoses in persons with early to moderate dementia and sleep disturbance. Behav Sleep Med. 2010;8:48–61. 20043249 10.1080/15402000903425769 PMC3745281

[6] Rose KM, Beck C, Tsai PF, Sleep disturbances and nocturnal agitation behaviors in older adults with dementia. Sleep. 2011;34:779–786. 21629366 10.5665/SLEEP.1048 PMC3098946

[7] Richards KC, Bost JE, Rogers V, Diagnostic accuracy of behavioral, activity, ferritin, and clinical indicators of restless legs syndrome. Sleep. 2015;38:371–380. 25325464 10.5665/sleep.4492 PMC4335520

[8] Richards K, Morrison J, Wang YY, Nighttime agitation and restless legs syndrome in persons with Alzheimer’s disease: study protocol for a double-blind, placebo-controlled, randomized trial (NightRest). Res Gerontol Nurs. 2020;13:280–288. 32966585 10.3928/19404921-20200918-01 PMC9112187

[9] U.S. Food and Drug Administration. FDA approved labeling texts dated December 26, 2012; May 1, 2013; April 2, 2020; April 17, 2020. Drugs@FDA. Published online. Accessed August 16, 2024. https://www.accessdata.fda.gov

[10] Yaltho TC, Ondo WG. The use of gabapentin enacarbil in the treatment of restless legs syndrome. Ther Adv Neurol Disord. 2010;3:269–275. 21179617 10.1177/1756285610378059 PMC3002665

[11] Parmelee PA, Thuras PD, Katz IR, Lawton MP. Validation of the cumulative illness rating scale in a geriatric residential population. J Am Geriatr Soc. 1995; 43:130–137. 7836636 10.1111/j.1532-5415.1995.tb06377.x

[12] Chrisman M, Tabar D, Whall AL, Booth DE. Agitated behavior in the cognitively impaired elderly. J Gerontol Nurs. 1991;17:9–13. 10.3928/0098-9134-19911201-04 1761822

[13] Finkel S, Lyons JS, Anderson RL. Reliability and validity of the Cohen-Mansfield agitation inventory in institutionalized elderly. Int J Geriatr Psychiatry. 1992;7: 487–490.

[14] Cohen-Mansfield J Instruction manual for the Cohen-Mansfield Agitation Inventory (CMAI). Rockville, MD: Research Institute of the Hebrew Home of Greater Washington; 1991.

[15] Schneider LS, Olin JT, Doody RS, Validity and reliability of the Alzheimer’s disease cooperative study-clinical global impression of change. Alzheimer Dis Assoc Disord. 1997;11(Suppl 2):S22–S32. 10.1097/00002093-199700112-00004 9236949

[16] Pigott TD. A review of methods for missing data, educational research and evaluation. Educ Res Eval. 2001;7:353–383.

[17] Jakobsen JC, Gluud C, Wetterslev J, Winkel P. When and how should multiple imputation be used for handling missing data in randomized clinical trials - a practical guide with flowcharts. BMC Med Res Methodol. 2017;17:1–10. 29207961 10.1186/s12874-017-0442-1 PMC5717805

[18] Little RJA, Rubin DB. Complete-case and available-case analysis, including weighting methods. In: Little RJA, Rubin DB, eds. Statistical Analysis with Missing Data. 2nd ed. Wiley; 2002.

[19] Meunier J, Creel K, Loubert A, Defining a clinically meaningful within-patient change threshold for the Cohen-Mansfield Agitation Inventory in Alzheimer’s dementia. Front Neurol. 2024;15:1379062. 39108660 10.3389/fneur.2024.1379062 PMC11301780

[20] De Crescenzo F, D’Alò GL, Ostinelli EG, Comparative effects of pharmacological interventions for the acute and long-term management of insomnia disorder in adults: a systematic review and network meta-analysis. Lancet. 2022;400:170–184. 35843245 10.1016/S0140-6736(22)00878-9

[21] Baron RM, Kenny DA. The moderator-mediator variable distinction in social psychological research: conceptual, strategic, and statistical considerations. J Pers Soc Psychol. 1986;51:1173–1182. 3806354 10.1037//0022-3514.51.6.1173

[22] Muller D, Judd CM, Yzerbyt Y. When moderation is mediated and mediation is moderated. J Pers Soc Psychol. 2005;89:852–863. 16393020 10.1037/0022-3514.89.6.852

[23] Stillhart C, Asteriadis A, Bocharova E, The impact of advanced age on gastrointestinal characteristics that are relevant to oral drug absorption: an AGePOP review. Eur J Pharm Sci. 2023;187:106452. 37098371 10.1016/j.ejps.2023.106452

[24] Lee D, Slomkowski M, Hefting N, Brexpiprazole for treatment of agitation in Alzheimer’s dementia. JAMA Neurol. 2023;80:1307–1316. 37930669 10.1001/jamaneurol.2023.3810 PMC10628834

[25] McQueen D, Cohen S, St John-Smith P, Rampes H. Rethinking placebo in psychiatry: how and why placebo effects occur. Adv Psychiatr Treat. 2013;19: 171–180.

[26] Pistacchi M, Gioulis M, Contin F, Sleep disturbance and cognitive disorder: epidemiological analysis in a cohort of 263 patients. Neurol Sci. 2014;35: 1955–1962. 25034185 10.1007/s10072-014-1870-x

[27] Allen RP, Montplaisir J, Walters A, Hogl B, Ferini-Strambi L. Restless legs syndrome (Willis-Ekbom Disease) and periodic limb movements during sleep. In: Kryger M, Roth T, Goldstein CA, Dement WC, eds. Principles and Practice of Sleep Medicine. 7th ed. Elsevier; 2022. p. 1119–1131.

