# Supplementary material for: Treatment of Restless Legs Syndrome Improves Agitation and Sleep in Persons with Dementia: A Randomized Trial
Source: J Am Med Dir Assoc. Author manuscript; Available in PMC 2026 Jun 5. (PMC13238233; doi:10.1016/j.jamda.2025.105485)
Supplement: 1 [file NIHMS2170211-supplement-1.docx]

**Supplemental Table 1. Mixed Model of Agitation Behaviors Measured using Direct Observation and the Cohen-Mansfield Agitation Inventory, Imputed Data**

| **Dependent** | **Predictor** | **Treatment Group** | **Week** | **Estimate (95% CI)** | **Individual *P* value^*^** | **Overall *P* value^†^** |
| --- | --- | --- | --- | --- | --- | --- |
| **Agitation Behaviors** |  |  |  |  |  |  |
| CMAI‡ (5-10 pm) | Week (REF**=0) |  | 2 | -3.22 (-8.70, 2.26) | .249 | .001 |
|  |  |  | 8 | -4.16 (-9.64, 1.32) | .136 |  |
|  | Treatment Group (REF=Placebo) | GEn§ |  | 6.24 (-1.89, 14.38) | .132 | .280 |
|  | Treatment Group (REF=Placebo)*Week (REF=0) | GEn | 2 | -1.38 (-9.16, 6.40) | .728 | .263 |
|  |  | GEn | 8 | -6.17 (-13.95, 1.61) | .120 |  |
|  | Mean Arterial Pressure |  |  | -0.26 (-0.60, 0.09) | .147 | .147 |
| CMAI (10 pm-7 am) | Week (REF=0) |  | 2 | -2.47 (-10.70, 5.76) | .555 | <.001 |
|  |  |  | 8 | -8.20 (-16.43, 0.03) | .051 |  |
|  | Treatment Group (REF=Placebo) | GEn |  | 13.34 (3.28, 23.39) | .010 | .595 |
|  | Treatment Group (REF=Placebo)*Week (REF=0) | GEn | 2 | -17.74 (-29.42, -6.07) | .003 | .005 |
|  |  | GEn | 8 | -16.23 (-27.90, -4.55) | .007 |  |
|  | Mean Arterial Pressure |  |  | 0.27 (-0.11, 0.65) | .163 | .163 |
| CMAI (5 pm –  7 am) Total | Week (REF=0) |  | 2 | -5.69 (-15.73, 4.35) | .266 | <.001 |
|  |  |  | 8 | -12.36 (-22.41, -2.32) | .016 |  |
|  | Treatment Group (REF=Placebo) | GEn |  | 19.58 (6.07, 33.10) | .005 | .293 |
|  | Treatment Group (REF=Placebo)*Week (REF=0) | GEn | 2 | -19.12 (-33.37, -4.87) | .009 | .004 |
|  |  | GEn | 8 | -22.39 (-36.64, -8.14) | .002 |  |
|  | Mean Arterial Pressure |  |  | 0.01 (-0.54, 0.56) | .958 | .958 |
| **Agitation Behaviors Index**\|\| | | |  |  |  |  |
| CMAI Index (5-10 pm) | Week (REF=0) |  | 2 | -0.62 (-1.78, 0.55) | .299 | .004 |
|  |  |  | 8 | -0.89 (-2.05, 0.28) | .135 |  |
|  | Treatment Group (REF=Placebo) | GEn |  | 1.27 (-0.49, 3.03) | .156 | .348 |
|  | Treatment Group (REF=Placebo)*Week (REF=0) | GEn | 2 | -0.68 (-2.33, 0.97) | .420 | .468 |
|  |  | GEn | 8 | -1.02 (-2.67, 0.63) | .226 |  |
|  | Mean Arterial Pressure |  |  | -0.06 (-0.14, 0.02) | .117 | .117 |
| CMAI Index (10 pm – 7am) | Week (REF=0) |  | 2 | -0.34 (-1.35, 0.66) | .503 | <.001 |
|  |  |  | 8 | -0.98 (-1.98, 0.03) | .056 |  |
|  | Treatment Group (REF=Placebo) | GEn |  | 1.66 (0.45, 2.87) | .007 | .633 |
|  | Treatment Group (REF=Placebo)*Week (REF=0) | GEn | 2 | -2.21 (-3.64, -0.79) | .002 | .003 |
|  |  | GEn | 8 | -2.13 (-3.55, -0.70) | .004 |  |
|  | Mean Arterial Pressure |  |  | 0.04 (-0.00, 0.09) | .071 | .071 |
| CMAI Total (5 pm – 7 am) Index | Week (REF=0) |  | 2 | -0.51 (-1.28, 0.27) | .201 | <.001 |
|  |  |  | 8 | -0.95 (-1.73, -0.18) | .016 |  |
|  | Treatment Group (REF=Placebo) | GEn |  | 1.45 (0.41, 2.50) | .007 | .326 |
|  | Treatment Group (REF=Placebo)*Week (REF=0) | GEn | 2 | -1.45 (-2.55, -0.35) | .010 | .006 |
|  |  | GEn | 8 | -1.67 (-2.77, -0.57) | .003 |  |
|  | Mean Arterial Pressure |  |  | 0.004 (-0.04, 0.05) | .870 | .870 |
|  | | | | | | |

NOTE. ^*^*P* values are based on tests of the estimates of fixed effects; ^†^*P* values are based on Type 3 tests of fixed effects; ‡CMAI Agitation Behaviors: Cohen-Mansfield Agitation Inventory, total # of behaviors observed during interval; §GEn: gabapentin enacarbil; ||CMAI Index: number of behaviors per hour of observation, range 0-168 per hour of observation; **REF: reference.

**Supplemental Table 2. Mixed Model of Sleep Disturbance with Imputed or Observed Data**

| **Dependent** | **Predictor** | **Treatment Group** | **Week** | **Estimate (95% CI)** | **Individual *P* value^*^** | **Overall *P* value^†^** |
| --- | --- | --- | --- | --- | --- | --- |
| **Sleep Actigraphy Measures** | |  |  |  |  |  |
| Total Nighttime Sleep, (minutes)^‡^ | Week (REF§=0) |  | 2 | 5.44 (-21.58, 32.46) | .691 | .001 |
|  |  |  | 8 | 14.87 (-14.24, 43.99) | .314 |  |
|  | Treatment Group (REF=Placebo) | GEn\|\| |  | -26.97 (-81.53, 27.60) | .330 | .990 |
|  | Treatment Group (REF=Placebo)*Week (REF=0) | GEn | 2 | 33.39 (-5.44, 72.21) | .091 | .062 |
|  |  | GEn | 8 | 48.45 (5.94, 90.96) | .026 |  |
|  | Mean Arterial Pressure |  |  | 0.96 (-1.75, 3.67) | .485 | .485 |
| Total Daytime Sleep, (minutes)^‡^ | Week (REF=0) |  | 2 | 13.90 (-7.47, 35.26) | .200 | .005 |
|  |  |  | 8 | 15.28 (-7.74, 38.30) | .191 |  |
|  | Treatment Group (REF=Placebo) | GEn |  | 20.96 (-24.02, 65.94) | .358 | .230 |
|  | Treatment Group (REF=Placebo)*Week (REF=0) | GEn | 2 | -10.23 (-40.89, 20.44) | .510 | .104 |
|  |  | GEn | 8 | 25.80 (-7.77, 59.38) | .131 |  |
|  | Mean Arterial Pressure |  |  | -0.19 (-2.44, 2.06) | .866 | .866 |
| Wake after Sleep Onset, (minutes)^‡^ | Week (REF=0) |  | 2 | -19.14 (-41.30, 3.02) | .090 | .092 |
|  |  |  | 8 | -14.40 (-38.24, 9.44) | .234 |  |
|  | Treatment Group (REF=Placebo) | GEn |  | -18.01 (-52.30, 16.27) | .301 | .392 |
|  | Treatment Group (REF=Placebo)*Week (REF=0) | GEn | 2 | 2.82 (-29.00, 34.65) | .861 | .826 |
|  |  | GEn | 8 | 10.72 (-24.06, 45.50) | .543 |  |
|  | Mean Arterial Pressure |  |  | 0.16 (-1.46, 1.79) | .841 | .841 |
| Nighttime Sleep efficiency^‡^ (percent) | Week (REF=0) |  | 2 | 0.76 (-2.99, 4.50) | .690 | .001 |
|  |  |  | 8 | 2.06 (-1.98, 6.10) | .314 |  |
|  | Treatment Group (REF=Placebo) | GEn |  | -3.74 (-11.32, 3.83) | .330 | .991 |
|  | Treatment Group (REF=Placebo)*Week (REF=0) | GEn | 2 | 4.63 (-0.76, 10.01) | .091 | .062 |
|  |  | GEn | 8 | 6.72 (0.82, 12.62) | .026 |  |
|  | Mean Arterial Pressure |  |  | 0.13 (-0.24, 0.51) | .486 | .486 |
| Nighttime Sleep Latency^‡^ (minutes) | Week (REF=0) |  | 2 | 11.40 (-9.87, 32.66) | .291 | .301 |
|  |  |  | 8 | 1.40 (-21.45, 24.25) | .903 |  |
|  | Treatment Group (REF=Placebo) | GEn |  | 12.70 (-17.02, 42.42) | .399 | .569 |
|  | Treatment Group (REF=Placebo)*Week (REF=0) | GEn | 2 | -5.13 (-35.66, 25.40) | .740 | .826 |
|  |  | GEn | 8 | -10.34 (-43.66, 22.98) | .540 |  |
|  | Mean Arterial Pressure |  |  | -0.77 (-2.13, 0.60) | .267 | .267 |
| **Sleep Disturbance – Direct Observation** | | |  |  |  |  |
| Wake Observed by RA** (number) | Week (REF=0) |  | 2 | -1.51 (-7.82, 4.79) | .637 | <.001 |
|  |  |  | 8 | -4.93 (-11.24, 1.38) | .125 |  |
|  | Treatment Group (REF=Placebo) | GEn |  | 8.02 (-0.92, 16.96) | .079 | .972 |
|  | Treatment Group (REF=Placebo)*Week (REF=0) | GEn | 2 | -12.54 (-21.49, -3.59) | .006 | .011 |
|  |  | GEn | 8 | -11.12 (-20.07, -2.17) | .015 |  |
|  | Mean Arterial Pressure |  |  | 0.08 (-0.29, 0.45) | .675 | .675 |
|  | | | | | | |

NOTE. ^*^*P* values are based on tests of the estimates of fixed effects; ^†^*P* values are based on Type 3 tests of fixed effects**;** ‡ For actigraphy variables total nighttime sleep, wake after sleep onset, nighttime sleep efficiency, and daytime sleep, statistics were based on complete case analysis; §REF: reference; ||GEn: gabapentin enacarbil; **RA: research assistant.

**Supplemental Table 3. Mixed Model of CMAI Caregiver and RLS Behaviors with Imputed Data**

| **Dependent** | **Predictor** | **Treatment Group** | **Week** | **Estimate (95% CI)** | **Individual *P* value^*^** | **Overall *P* value^†^** |
| --- | --- | --- | --- | --- | --- | --- |
| ‡CMAI Caregiver | Week (REF**=0) |  | 2 | 0.11 (-2.44, 2.65) | .935 | .074 |
|  |  |  | 8 | -1.90 (-4.44, 0.65) | .143 |  |
|  | Treatment Group (REF=Placebo) | GEn |  | -0.12 (-4.03, 3.80) | .954 | .483 |
|  | Treatment Group (REF=Placebo)*Week (REF=0) | GEn | 2 | -2.84 (-6.45, 0.77) | .123 | .244 |
|  |  | GEn | 8 | -0.36 (-3.97, 3.25) | .844 |  |
|  | Mean Arterial Pressure |  |  | 0.01 (-0.16, 0.18) | .909 | .909 |
| RLS\|\| Behaviors (raw score, number) | Week (REF=0) |  | 2 | -2.86 (-4.73, -1.00) | 0.003 | <.001 |
|  |  |  | 8 | -3.38 (-5.24, -1.52) | <.001 |  |
|  | Treatment Group (REF=Placebo) | GEn |  | -1.10 (-3.71, 1.52) | .410 | .603 |
|  | Treatment Group (REF=Placebo)*Week (REF=0) | GEn | 2 | 1.66 (-0.99, 4.30) | .218 | .353 |
|  |  | GEn | 8 | -0.05 (-2.69, 2.59) | .970 |  |
|  | Mean Arterial Pressure |  |  | 0.01 (-0.10, 0.12) | .847 | .847 |

NOTE. ^*^*P* values are based on tests of the estimates of fixed effects; ^†^*P* values are based on Type 3 tests of fixed effects**;** ‡CMAI: Cohen Mansfield Agitation Inventory; §GEn: gabapentin enacarbil; ||RLS: restless legs syndrome; **REF: reference.

**Supplemental Table 4. Incidence and Consequence of Falls in Randomized Participants (N=147)**

| **Incidence of Falls**  **(# of Falls per Category)/**  **(Total # of Falls=145)** | **Total N** | **Consequence of Fall†** | | | **P-value** |
| --- | --- | --- | --- | --- | --- |
|  |  | **None**  **N (%)** | **Minor-Moderate**  **N (%)** | **Major**  **N (%)** |  |
| *All falls, N | 145 | 108 (74.5%) | 33 (22.8%) | 4 ( 2.8%) | .770 |
| GEn‡ | 93 | 69 (74.2%) | 22 (23.7%) | 2 ( 2.2%) |  |
| Placebo | 52 | 39 (75.0%) | 11 (21.2%) | 2 ( 3.8%) |  |
|  | | | | | |

NOTE.; *Total number of falls (Participants may have more than one fall); †Consequence of Fall - None: Participant had no injuries (signs or symptoms) resulting from the fall. If an x-ray or scan or other post fall evaluation - also results in a finding of no injury. Minor/Moderate: Participant required dressing, ice, limb elevation, suturing, skin glue, splinting, muscle/joint strain. Major: Participant required surgery, casting, traction, consultation sought for neurological/internal injury, or for participants with coagulopathy, administration of blood products; ‡GEn: gabapentin enacarbil

**Supplemental Table 5. Number of Falls in Randomized Participants (N=147)**

|  | **Overall**  **N = 147** | **GEn †**  **N = 74** | **Placebo**  **N = 73** | **P-value*** |
| --- | --- | --- | --- | --- |
| **Number of Falls** |  |  |  | .030 |
| Mean | 1.0 | 1.3 | 0.7 |  |
| SD‡ | 1.6 | 1.9 | 1.2 |  |
| Median | 0 | 1 | 0 |  |
| IQR (Q1, Q3) | (0, 1) | (0, 2) | (0, 1) |  |
| Range (Min, Max)\|\| | (0, 11) | (0, 11) | (0, 7) |  |
| Number of Falls, n (%) |  |  |  | .246 |
| 0 | 76 (51.7%) | 32 (43.2%) | 44 (60.3%) |  |
| 1 | 41 (27.9%) | 23 (31.1%) | 18 (24.7%) |  |
| 2 | 14 (9.5%) | 9 (12.2%) | 5 (6.9%) |  |
| 3 | 5 (3.4%) | 3 (4.1%) | 2 (2.7%) |  |
| 4 | 5 (3.4%) | 3 (4.1%) | 2 (2.7%) |  |
| 5+ | 6 (4.1%) | 5 (6.8%) | 1 (1.4%) |  |

NOTE. *P-values based on non-parametric Wilcoxon rank-sum and Fisher’s exact tests, as appropriate.  †GEn: gabapentin enacarbil; ‡SD: Standard Deviation; §IQR: Interquartile Range; Q1: 1^st^ Quartile; 3^rd^ Quartile; ||Min: Minimum; Max: Maximum.
